# The Antidepressant-Like Effects of a Clinically Relevant Dose of Ketamine Are Accompanied by Biphasic Alterations in Working Memory in the Wistar Kyoto Rat Model of Depression

**DOI:** 10.3389/fpsyt.2020.599588

**Published:** 2021-01-20

**Authors:** Conor W. McDonnell, Fionn Dunphy-Doherty, Jennifer Rouine, Massimiliano Bianchi, Neil Upton, Ewa Sokolowska, Jack A. Prenderville

**Affiliations:** ^1^Transpharmation Ireland Ltd., Trinity College Institute of Neuroscience (TCIN), Trinity College Dublin, Dublin, Ireland; ^2^Transpharmation Ltd., London Biosciences Innovation Centre, London, United Kingdom

**Keywords:** animal model, antidepressant, ketamine, plasma exposure, working memory, Wistar Kyoto, depression

## Abstract

Major depressive disorder (MDD) is the leading cause of disability worldwide. The majority of antidepressant drugs require several weeks or months of treatment to demonstrate efficacy and a subset of patients are resistant to such interventions. Ketamine demonstrates rapid and long-lasting antidepressant effects in treatment resistant patients; however, side effects may limit its widespread clinical utility. The pharmaceutical industry is engaged in developing novel rapid-acting antidepressant drugs and the establishment of clinically relevant assays are needed to advance this process. Wistar Kyoto (WKY) rats are a valuable model of many of the characteristics of MDD and their resistance to selective serotonin reuptake inhibitors (SSRIs) in several behavioral paradigms emulates treatment resistance in clinical populations. Here, we confirmed the depressive-like phenotype of WKY rats in comparison to Sprague Dawley rats, characterized by increased immobility in the forced swim test, decreased locomotor activity and entries to the centre in the open field test, anhedonia in the female urine sniffing test and working memory deficits in the delayed non-match to position task. Single subcutaneous administration of 5 mg/kg ketamine in WKY rats mirrored the plasma exposure produced by the antidepressant dose in the clinic and rescued depressive-like behaviors. The same dose induced transient side effects, including decreased locomotor activity and reduced positive affect-associated vocalizations. Furthermore, ketamine acutely impaired working memory but induced pro-cognitive effects at a later time point. These data confirm the WKY rat as a preclinical model of depression. Ketamine's efficacy in recovering this depressive-like phenotype while inducing transient dissociative-like effects supports this as a translational model suitable for investigating novel antidepressant drugs.

## Introduction

The estimated lifetime prevalence of major depressive disorder (MDD) in Europe is 12.8% (1). The majority of individuals diagnosed with MDD seek treatment (2) and the estimated European 12-month prevalence of antidepressant use is 7.2% (3). Most antidepressant drugs on the market, including selective serotonin reuptake inhibitors (SSRIs), target monoamine neurotransmission and often take weeks or months to demonstrate a therapeutic effect (4). Furthermore, many patients do not achieve remission following antidepressant treatment, with some estimates indicating ~35% do not respond to two different pharmacological interventions (5), a subtype of the disease often described as treatment resistant depression (TRD).

A single sub-anesthetic dose of the non-competitive *N*-methyl-*D*-aspartate (NMDA) receptor antagonist ketamine produces rapid (within hours) and long lasting (up to 7 days) antidepressant effects in TRD patients (6). Recently, its *S*-enantiomer, esketamine, has shown efficacy as an adjunct therapy in TRD (7), receiving approval for use in combination with an oral antidepressant in 2019. Despite this, and the growing clinical and preclinical evidence of ketamine efficacy in mood disorders (8), its widespread clinical utility may be limited by abuse potential (9) and side-effects, including dissociation, cognitive impairment and psychosis (10, 11). Therefore, the pharmaceutical industry is actively engaged in developing novel drugs having ketamine-like efficacy with the aim of identifying rapid-acting antidepressant drugs with minimal side-effects (12, 13). The identification of translational assays to model the effects of ketamine preclinically are required to advance this drug discovery process.

Wistar Kyoto (WKY) rats are proposed as a model of MDD, displaying a behavioral and physiological phenotype comparable to depressed patients (14). When compared to other strains, WKY rats show increased immobility in the forced swimming test (FST), considered an index of depressive-like behavior (15, 16). Immobility in the FST is reduced by antidepressant drugs (17). WKY rats are an exception to this, showing responsiveness to tricyclic antidepressants (TCAs) but resistance to SSRIs (16, 18, 19). Consequently, WKY rats were hypothesized to be a model of antidepressant resistance that may be appropriate for studying TRD (18, 20). This is strengthened by evidence of antidepressant-like effects of sub-anesthetic doses of ketamine in female WKY rats (21).

In addition to the FST, progress has been made in developing translational assays to model symptoms of MDD. The female urine sniffing test (FUST) has been proposed as a method to assess hedonic behavior and therefore anhedonia in rodents (22). Ultrasonic vocalizations (USVs) emitted by rodents have been suggested as a method for assessing affective state, either alone (23) or in combination with the FUST (22). USVs are also increased during social interactions and are linked to communication (24). Furthermore, impaired cognitive function, and in particular a deficit in working memory, is a well-recognized symptom of MDD (25, 26). There are insufficient treatment options for such cognitive symptoms of MDD (27) and this is compounded by a limited availability of translational preclinical models facilitating drug development (28).

Here, we first assess the behavioral phenotype of WKY rats, evaluating anxiety-like behavior in the open field test, depressive-like behavior in the FST, in addition to clinically relevant behaviors such as anhedonia and working memory function. We explore the dose-response efficacy of low dose ketamine in the FST in WKY rats and identify a dosing regimen that replicates the plasma drug exposure induced by the clinical antidepressant dose of ketamine. Using this dosing regimen, we investigate the time course of the antidepressant-like efficacy of ketamine, and temporal effects on locomotor activity (LMA), affective state-associated USVs and working memory.

## Materials and Methods

### Animals and Experimental Protocol

Adult male Sprague Dawley (SD; *n* = 45, 200–250 g), and Wistar Kyoto (WKY; *n* = 203, 150–200 g; Envigo Correzzana, Italy and Blackthorn, England) were group housed (2–4 per cage) with environmental enrichment and maintained on a standard 12 h light/dark cycle with food/water available *ad libitum*. Experimental procedures started after 2 weeks acclimatization to the environment. The experimental procedures were in accordance with the European Communities Council Directive (Directive 2010/63/EU into the Irish SI 543/2012).

Experiment 1: Behavioral differences between drug-free SD and WKY rats.

Behavioral assays were performed to investigate: (i) anxiety-like behavior and locomotor activity (LMA; *n* = 7–8 per group); (ii) depressive-like behavior in the FST (*n* = 10 per group); (iii) hedonic behavior in the FUST (*n* = 8–10 per group); (iv) non-evoked USVs recorded in the home cage of pair housed rats (*n* = 5 pairs of rats); (v) working memory in the delayed non-match to position task (DNMP; *n* = 9–10 per group).

Experiment 2: Dose-response efficacy of acute ketamine in the FST and plasma/brain drug exposure analysis.

The dose-response efficacy of acute ketamine (1, 3, or 5 mg/kg) administered by subcutaneous (s.c.) injection (29), on depressive-like behavior in WKY rats (*n* = 10 per group) was tested in the FST 30 min post-administration. To analyse plasma drug concentration *n* = 3 from each ketamine treatment group were euthanised immediately after the FST (36 min after ketamine administration).

In a separate group of rats, plasma drug concentration was analyzed 5, 10, 15, 30, 60, 240, and 360 min after ketamine (5 mg/kg, s.c.) administration (*n* = 3 per time point). The concentration of ketamine in the brain was analyzed at 15, 60, 240, and 360-min post-administration (*n* = 3 per time point).

Experiment 3: Time-course efficacy of acute ketamine in the FST, emission of USVs and DNMP task.

WKY rats received a single administration of ketamine (5 mg/kg, s.c.) or vehicle and depressive-like behavior was tested in the FST 30 min and 24 h post-administration, separate groups of rats were used for each time point (*n* = 10 per group). LMA was assessed at the same time points in separate groups of rats (*n* = 8–9 per group).

The number of 50 kHz USVs emitted by a pair of WKY rats was recorded in naïve animals (no injection), and vehicle and ketamine (5 mg/kg, s.c.) treated animals at 30 min and 24 h post-administration (*n* = 5 pairs).

The DNMP task was employed to investigate working memory at the same time points in a separate group of rats (*n* = 9–10) after a single administration of ketamine (5 mg/kg, s.c.). SD rats administered vehicle solution were used as the control group.

### Open Field Test/LMA

LMA was assessed in an open field (50cm height, 100 cm diameter) and recorded by camera above the arena. The 5 min sessions were analyzed using Ethovision XT 11 (Noldus, The Netherlands). Distance traveled (cm) and number of entries to the center were analyzed. Frequency of entries to centre of the arena were used as a measure of anxiety-like behavior (30).

### FUST

The FUST was used to assess reward-seeking behavior and was performed as previously described (22). Rats were singly housed for 1 week prior to the experiment. One hour before the test a sterile cotton tip was inserted into the home cage to habituate the animals. Testing was performed in the home cage and rats were transferred to the testing room (3 lux lighting) immediately after habituation. For the test rats were first exposed to a cotton tip infused with 60 μl sterile water for 3 min. After an interval of 45 min rats were exposed to a new cotton tip infused with 60 μl female urine collected during oestrus from rats of the same strain for 3 min. After an interval of 45 min rats were exposed to a new cotton tip infused with 60 μl male urine collected from rats of the same strain for 3 min. The test session was recorded using a video camera placed above the cage. The time(s) spent sniffing water and urine was measured by an experimenter blind to treatment groups.

### USV Recording

USV recording was performed using SONOTRACK 1.5.0 (Metris B.V.) with a single microphone (15–125 kHz) placed 30 cm above the cage. USVs at 50 kHz were automatically counted using the SONOTRACK software and this was verified by manual counting of 50 kHz calls from the spectrogram. During the FUST USVs were recorded during the water and urine exposure phases.

USVs in all other experiments were recorded from pairs of rats in their home cage, a common method for measuring non-evoked USVs (24). In the case of these experiments using drug treatments each pair received the same drug. A group of rats receiving no injection was used to examine the effect of injection on USVs. The home cage environment included environmental enrichment.

### FST

The modified rat FST was used. Briefly, rats were individually placed into a glass cylinder (50 cm height, 20 cm diameter) containing 30 cm of water (25 ± 1°C) for a 5 min test session. Each test was recorded by camera above the cylinder. WKY rats exhibit spontaneous immobility in the FST, therefore no pre-test exposure to the FST is required as with other strains (19, 31). SD rats underwent the same FST protocol as WKY rats. Immobility(s) was measured when no activity was observed other than that required to keep the rat's head above the water and was used as a measure of depressive-like behavior (17). Two experimenters blind to treatment group performed the analysis and the mean values were used.

### DNMP Working Memory Task

Working memory was assessed using the DNMP task as previously described (32). DNMP was performed in an operant conditioning chamber (Med Associates; 30.5 × 24.1 × 21.0 cm). The front wall of the chamber contained two retractable levers (5.5 cm above the floor) separated by a 5.5 cm food hopper in the centre of the wall. The back wall contained one retractable lever 5.5 cm above the floor in the centre of the wall.

A single trial contained three phases. (i) Sample phase: the rat was required to press the left or right lever extended on a random basis from the front wall; (ii) Delay phase: after 1–30 s delay the rat was required to press the centre lever extended from the back wall; and (iii) Response phase: the left and right lever were extended on the front wall, a non-match response (i.e., press of the opposite lever to that presented during the sample phase) constitutes a correct response and a sucrose pellet (reinforcement) was delivered to food hopper.

Rats were habituated to the operant chambers and were rewarded on a continuous reinforcement schedule for responding to a lever inserted to the chamber. Following 8 days habituation non-match to sample training began. Rats completed 90 trials per day with 10 s inter-trial intervals. A single non-matching to sample trial is as described above with omission of delay phase. Rats were required to reach a criterion of 3 consecutive days of 85% correct responses before introduction of delay phase (i.e., initiation of the DNMP training). Rats were trained 5 days per week for 16 weeks before drug treatment. On each day, only rats that completed 90 trials were included in analysis.

**Figure 1:**
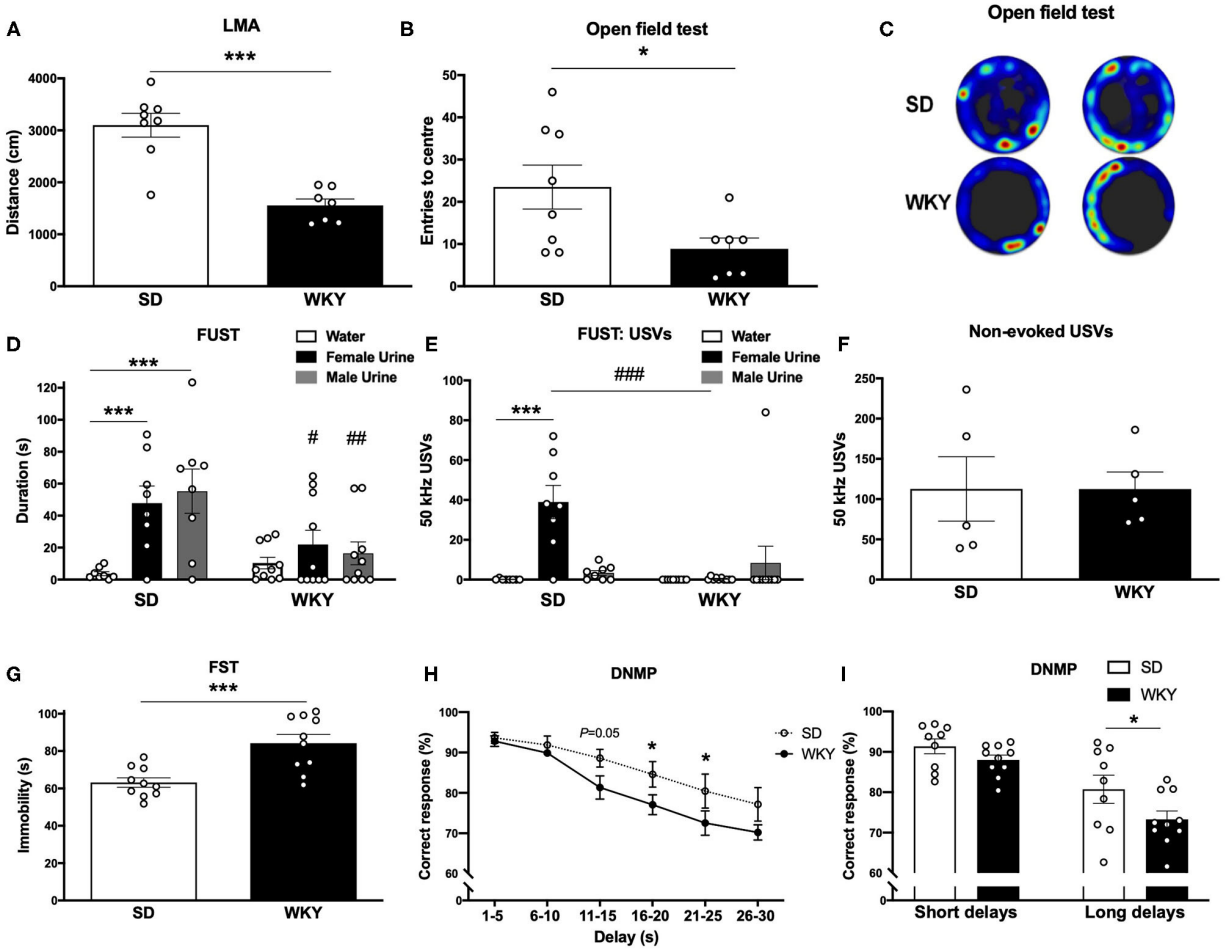
WKY rats showed a depressive-like phenotype in comparison to SD rats. **(A)** LMA (distance, cm) in OF test. **(B)** Entries to the centre in OF test. **(C)** Representative OF heat map from *n* = 2 SD and WKY rats. **(D)** Sniffing duration (s) in the FUST. **(E)** USVs: number of 50 kHz calls emitted during FUST. **(F)** Non-evoked 50 kHz calls recorded in the home cage. **(G)** Immobility in the FST. **(H)** Correct response in DNMP task stratified by delay period. **(I)** Correct responses in DNMP task grouped by short and long delay periods. Data are expressed as mean ± SEM, *n* = 5–10 per group. *p < 0.05, ***p < 0.001, #*p* < 0.05, ##*p* < 0.01, ###*p* < 0.001. WKY, Wistar Kyoto; SD, Sprague Dawley; LMA, locomotor activity; OF, open field test; FUST, female urine sniffing test; USVs, ultrasonic vocalizations; FST, forced swim test; DNMP, delayed non-match to position task.

In an initial experiment WKY and SD rats were compared to assess strain differences in working memory (experiment 1, Figure 1H-1). In this experiment an average of 5 consecutive days' performance was used to indicate “baseline” performance.

In a subsequent pharmacological experiment (experiment 3, **Figure 5**) WKY and SD rats were treated with vehicle solution and compared 30 min post-administration to confirm “baseline” performance. Following this WKY rats were treated with ketamine (5 mg/kg, s.c.) and performance in the task was evaluated 30 min and 24 h post-administration and compared to vehicle treated SD rats. The objective of this comparison was to explore the pharmacological effects of ketamine in the WKY rat model of depression relative to vehicle treated SD rats representing a control strain and receiving no drug.

### Ketamine

A stock solution (100 mg/ml) of ketamine hydrochloride (Animalcare Ltd., UK) was diluted in saline (0.9% NaCl) for s.c. injections in a volume of 1 ml/kg.

### Plasma and Brain Drug Exposure

Anesthesia was induced using isoflurane. Blood (400–500 μl) was collected by cardiac puncture (with thoracotomy) using a sterile syringe and transferred to Li-heparin tubes, manually agitated and stored on ice. Within 30 min of collection samples were centrifuged at 4°C, at 1,500 g, for 10 min to isolate plasma. Brain samples were homogenized in water using a Covaris ultra-focused homogeniser. Plasma and brain homogenate samples were protein precipitated using acetonitrile containing an internal standard. Ketamine was quantified by LC-MS/MS using a Waters Acquity UPLC coupled to a Waters TSQ mass spectrometer, operated in the positive ion electrospray mode. The UPLC system was operated using a Waters Acquity T3 column and running in gradient mode using acetonitrile and 1% formic acid in water.

### Statistical Analysis

Data was expressed as mean ± standard error of the mean (SEM). Student's *t*-test was used to analyse statistical difference between WKY and SD rats in Figures 1, **4**, and FST and LMA data in **Figure 3**. Two-way analysis of variance (ANOVA) was used to analyse FUST in Figure 1 and DNMP data in Figures 1, **5**. One-way ANOVA was used to analyse ketamine dose response in **Figure 3A** and USV data in **Figures 4B,C**. Fisher's least significant difference (LSD) was used for *post-hoc* analysis where appropriate. Values of *p* < 0.05 were considered statistically significant. Data was analyzed and graphs prepared using GraphPad Prism 8.

## Results

### WKY Rats Show Anxiety-Like Behavior, Anhedonia, Depressive-Like Behavior, and Working Memory Deficits Compared to SD Rats

Anxiety-like behavior was assessed in a novel environment open field test (Figures 1A–C), the FUST coupled to USVs recording was used to examine hedonic behavior (Figures 1D,E) and non-evoked USVs were recorded in the home cage of pair housed rats (Figure 1F). Depressive-like behavior was measured in FST (Figure 1G). Working memory was assessed in the DNMP task (Figures 1H,I). Separate groups of SD and WKY rats were used for each behavioral test.

WKY rats had reduced LMA (distance; cm) in the open field test compared to SD rats (*p* < 0.001, Student's *t*-test, *n* = 7–8 per group; Figure 1A). WKY rats had a reduced number of center entries compared to SD rats (*p* < 0.05, Student's *t*-test, *n* = 7–8 per group; Figures 1B,C).

SD and WKY rats (*n* = 8–10 per group) were exposed to three stimuli in the FUST: water, female urine and male urine; sniffing duration (s) was scored for each stimulus. The two-way ANOVA yielded a significant effect of stimulus (*F*_2, 48_ = 7.59, *p* < 0.01), strain (*F*_1, 48_ = 7.95, *p* < 0.01) and a significant stimulus x strain interaction (*F*_2, 48_ = 3.89, *p* < 0.05). *Post-hoc* analysis (Fisher's LSD test) revealed that SD rats spent significantly more time sniffing both female (*p* = 0.001) and male urine (*p* < 0.001) compared to water. WKY rats showed no preference for any stimulus. Compared to WKY rats SD rats spent significantly more time sniffing female urine (*p* < 0.05) and male urine (*p* < 0.01).

SD and WKY rats (*n* = 8–10 per group) were exposed to three stimuli in the FUST: water, female urine and male urine; USVs emitted during each stimulus were counted. The two-way ANOVA yielded a significant effect of stimulus (*F*_2, 48_ = 8.40, *p* < 0.001), strain (*F*_1, 48_ = 7.81, *p* < 0.01) and a significant stimulus x strain interaction (*F*_2, 48_ = 11.73, *p* < 0.001). *Post-hoc* analysis (Fisher's LSD test) revealed that SD rats emitted significantly more 50 kHz USVs during female urine exposure compared to water (*p* < 0.001) and male urine (*p* < 0.001). WKY rats showed no change in number of 50 kHz USVs emitted during exposure to any stimulus. In order to examine a potential strain difference in baseline 50 kHz USVs, SD and WKY rats were recorded in their home cage (*n* = 2 rats per cage, *n* = 5 cages per strain, Figure 1F). There was no significant difference in non-evoked 50 kHz USVs recorded from pairs of SD and WKY rats in their home cage.

Immobility in the FST was used as a measure of depressive-like behavior (*n* = 10 per group, Figure 1G). WKY rats showed a significant increase in immobility compared with SD rats (*p* < 0.001).

Working memory was assessed in the DNMP task (Figures 1H,I). WKY rats showed impaired DNMP performance compared to SD rats. A significant main effect of strain (two-way ANOVA, *F*_1, 102_ = 12.71, *p* < 0.001) and delay interval (two-way ANOVA, *F*_5, 102_ = 17.56, *p* < 0.001) was observed. There was no strain x delay interaction (two-way ANOVA, *F*_15, 102_ = 0.7126). *Post-hoc* analysis (Fisher's LSD test) revealed impairment performance at longer delay periods: 16–20 s (*p* < 0.05) and 21–25 s (*p* < 0.05) delays. WKY rats showed a trend toward an impairment at 11–15 s (*p* = 0.05). When delay periods were grouped into short (1–15 s) and long (16–20 s) delays a significant main effect of strain (two-way ANOVA, *F*_1, 34_ = 5.75, *p* < 0.05) and delay interval (two-way ANOVA, *F*_1, 34_ = 31.60, *p* < 0.001) was observed. There was no strain x delay interaction (two-way ANOVA, *F*_1, 34_ = 0.8267). *Post-hoc* analysis (Fisher's LSD test) revealed that WKY rats showed an impairment in correct responses in the long delay period (*p* < 0.05) in comparison to SD rats.

### Ketamine (5 mg/kg, s.c.) Administration in WKY Rats Produced Plasma Drug Exposure Similar to the Clinically Effective Dose

**Figure 2:**
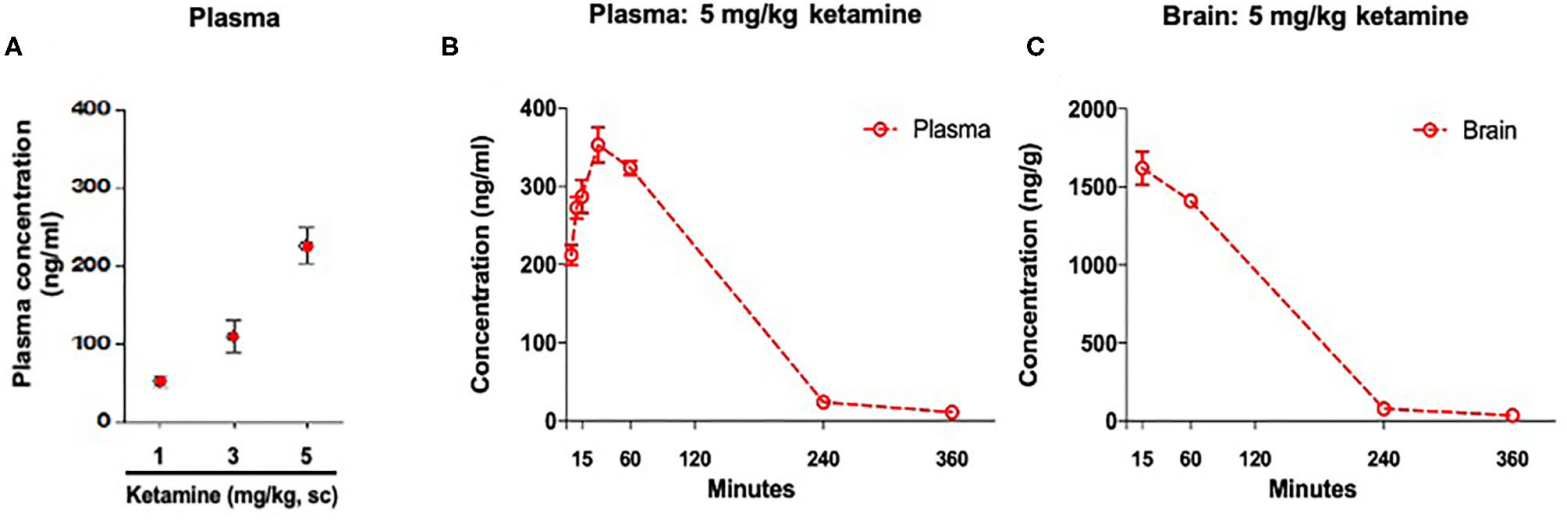
Concentration of ketamine in plasma and brain. **(A)** Plasma drug exposure 36 min post-administration of ketamine (1, 3, and 5 mg/kg, s.c.). **(B)** Time course of plasma drug exposure post-administration of ketamine 5 mg/kg, s.c. **(C)** Time course of brain drug exposure post-administration of ketamine 5 mg/kg, s.c. Data are expressed as mean ± SEM, *n* = 3 per group.

Plasma ketamine concentrations in WKY rats 36 min post-administration were: 51 ng/ml (1 mg/kg, s.c.), 108 ng/ml (3 mg/kg, s.c.), and 225 ng/ml (5 mg/kg, s.c.) (Figure 2A). An additional exposure study showed that at 30 min post-administration a single administration of ketamine (5 mg/kg, s.c.) produced a maximum plasma concentration of 353 ng/ml. A maximum brain concentration of 1,620 ng/g was observed 15 min post-administration with the same dose (Figures 2B,C).

### Ketamine Reduced Immobility in the FST in WKY Rats at 30 min and 24 h Post-administration

**Figure 3:**
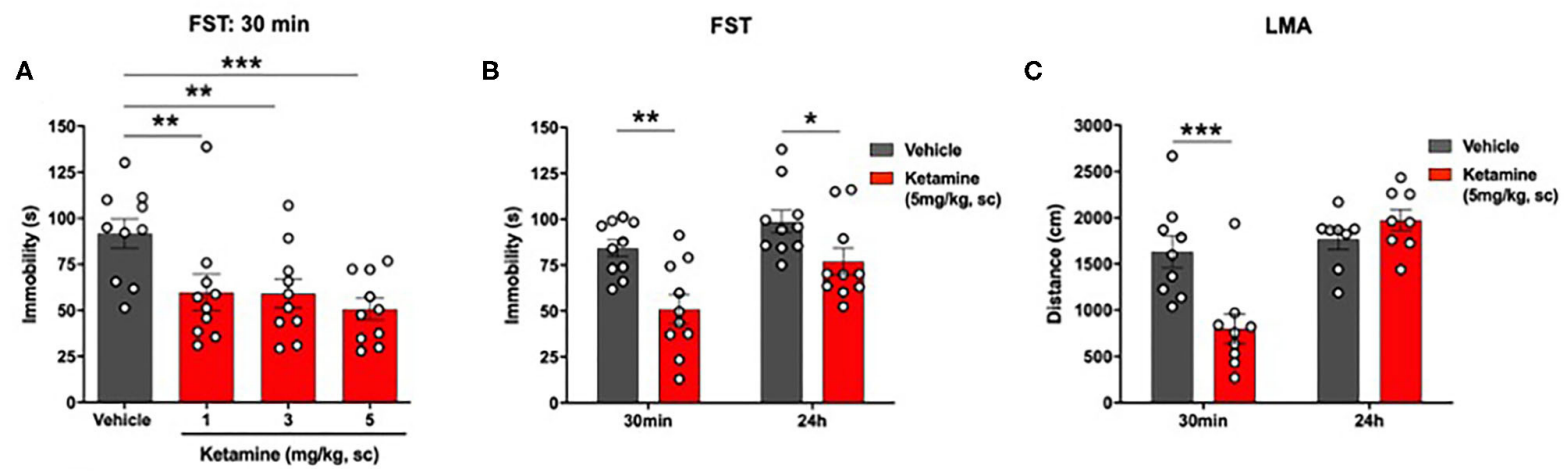
Single administration of ketamine produced a rapid (30 min) and sustained (24 h) reduction in immobility in the FST and transiently reduced LMA (30 min) in WKY rats. **(A)** Dose-response efficacy of acute ketamine (1, 3, and 5 mg/kg, s.c.) 30 min post-administration in the FST. **(B)** FST 30 min and 24 h post-administration of ketamine (5 mg/kg, s.c.). **(C)** LMA 30 min and 24 h post-administration of ketamine (5 mg/kg, s.c.). Data are expressed as mean ± SEM, *n* = 8-10 per group. *p < 0.05, **p < 0.01, ***p < 0.001. WKY, Wistar Kyoto; FST, forced swim test; LMA, locomotor activity.

Ketamine reduced immobility in the FST in WKY rats (*p* < 0.01, one-way ANOVA, *F*_3, 36_ = 5.14; Figure 3A). *Post-hoc* analysis (Fisher's LSD test) revealed a significant reduction in immobility at all 3 doses of ketamine, 1 mg/kg (*p* < 0.01), 3 mg/kg (*p* < 0.01), 5 mg/kg (*p* < 0.001) compared to vehicle.

A significant reduction in immobility in the FST was observed in the ketamine group compared to vehicle group at 30 min (*p* < 0.01, Student's *t*-test, Figure 3B) and 24 h (*p* < 0.01, Student's *t*-test; Figure 3B) post-administration.

### Ketamine Reduced LMA in WKY Rats at 30 min Post-administration Only

A significant reduction in LMA was observed in the ketamine treated group compared to vehicle 30 min post-administration (*p* < 0.01, Student's *t*-test; Figure 3C). Ketamine had no effect on LMA at 24 h.

### Ketamine Reduced Non-evoked USVs in WKY Rats 30 min Post-administration

**Figure 4:**
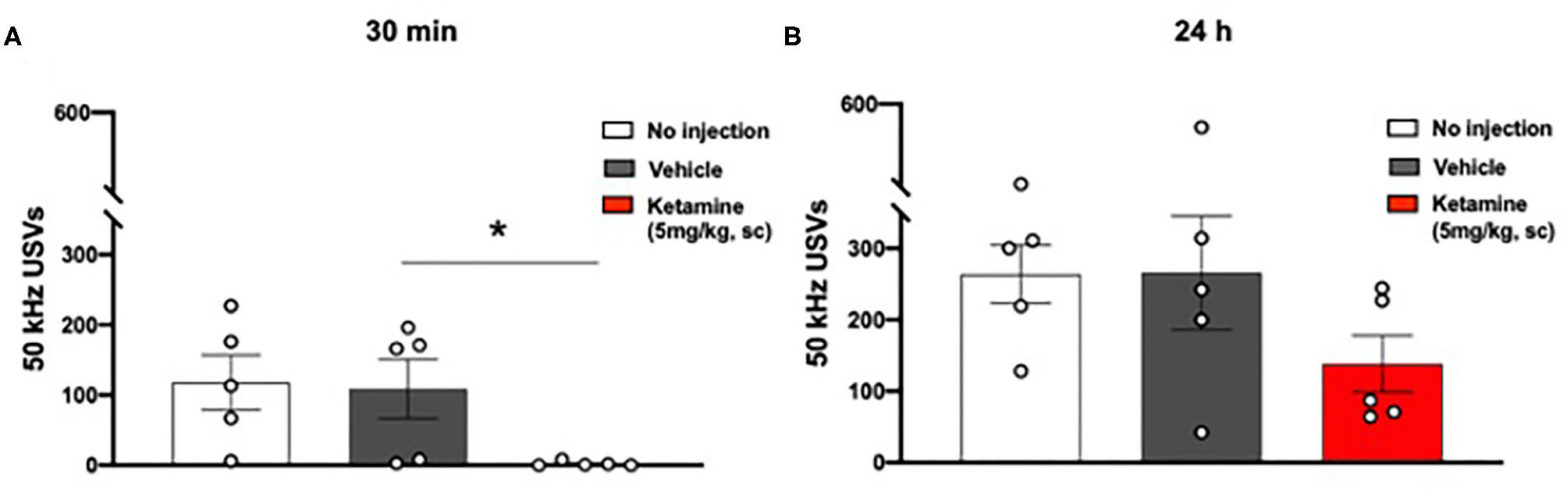
Single administration of ketamine transiently reduced positive affect associated 50 kHz USVs (30 min) in WKY rats. **(A)** USVs 30 min post-administration of ketamine (5 mg/kg, s.c.). **(B)** USVs 24 h post-administration of ketamine (5 mg/kg, s.c.). Data are expressed as mean ± SEM, *n* = 5 cages of rats; *n* = 2 rats per cage. *p < 0.05. WKY, Wistar Kyoto; USVs, ultrasonic vocalizations.

No significant overall effect of group on 50 kHz USVs 30 min after ketamine or vehicle administration was observed (*p* = 0.0551, one-way ANOVA, *F*_2, 12_ = 3.727, Figure 4A). *Post-hoc* analysis revealed a significant reduction in the number of 50 kHz USVs emitted by the ketamine treated group in comparison to the vehicle treated group (*p* < 0.05) and the group receiving no injection (*p* < 0.05).

No significant effect was observed 24 h after ketamine or vehicle administration (one-way ANOVA, *F*_2, 12_ = 1.653, Figure 4B).

### Ketamine Impaired Working Memory in WKY Rats at 30 min Post-administration

**Figure 5:**
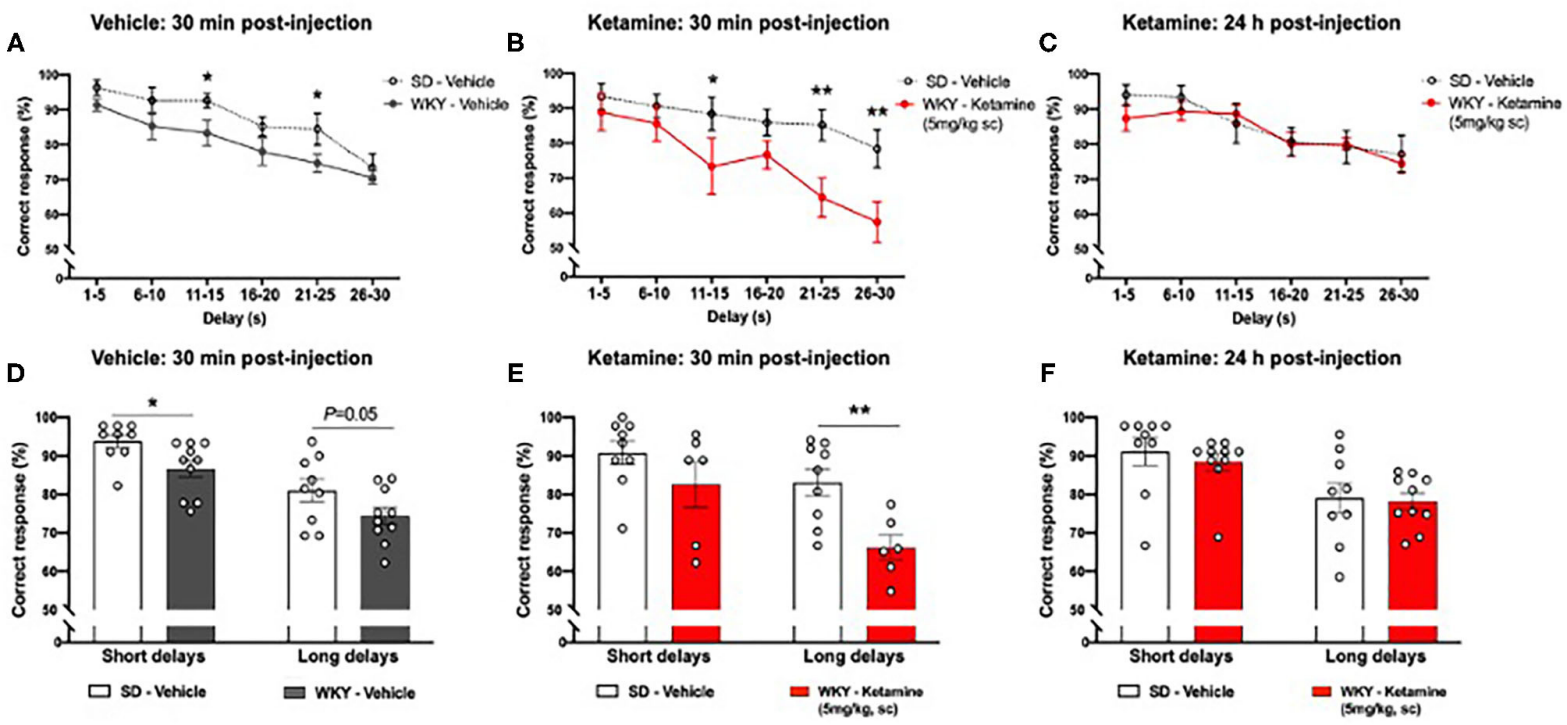
WKY showed impaired performance in the DNMP task in comparison to SD rats, single administration of ketamine further impaired performance 30 min post-administration but improved performance 24 h post-administration **(A)** WKY and SD performance in the DNMP 30 min after vehicle administration. **(B)** WKY 30 min post-administration of ketamine (5 mg/kg s.c.) and SD vehicle 30 min post-administration of vehicle. **(C)** WKY 24 h post-administration of ketamine (5 mg/kg, s.c.) and SD vehicle 24 h post-administration of vehicle. **(D)** WKY and SD performance 30 min after vehicle administration, correct responses grouped by short (1–15 s) and long (16–30 s) delay periods. **(E)** WKY 30 min post-administration of ketamine (5 mg/kg, s.c.) and SD vehicle 30 min post-administration of vehicle, correct responses grouped by short (1–15 s) and long (16–30 s) delay periods. **(F)** WKY 24 h post-administration of ketamine (5 mg/kg, s.c.) and SD vehicle 24 h post-administration of vehicle, correct responses grouped by short (1–15 s) and long (16–30 s) delay periods. Data are expressed as mean ± SEM, *n* = 9–10 per group. *p < 0.05, **p < 0.01. WKY, Wistar Kyoto; SD, Sprague Dawley; DNMP, delayed non-match to position task.

WKY rats showed impaired DNMP performance compared to SD rats following vehicle treatment (Figure 5A). A significant main effect of group (*p* < 0.001, two-way ANOVA, *F*_1, 102_ = 14.00), and delay interval (*p* < 0.001, two-way ANOVA, *F*_5, 102_ = 12.04) was observed. There was no strain x delay interaction (two-way ANOVA, *F*_5, 102_ = 0.3323). *Post-hoc* analysis (Fisher's LSD test) revealed impairment of performance at two delay intervals: 11–15 s (*p* < 0.05) and 21–25 s (*p* < 0.05). When delay periods were grouped into short (1–15 s) and long (16–20 s) delays (Figure 5D) a significant main effect of group (two-way ANOVA, *F*_1, 34_ = 8.773, *p* < 0.01) and delay interval (two-way ANOVA, *F*_1, 34_ = 29.00, *p* < 0.001) was observed. There was no strain x delay interaction (two-way ANOVA, *F*_1, 34_ = 0.013). *Post-hoc* analysis (Fisher's LSD test) revealed that WKY rats showed an impairment in correct responses in the short delay periods (*p* < 0.05) and a trend toward an impairment in the long delay periods (*p* = 0.05) in comparison to SD rats.

WKY rats showed further impairment in DNMP performance at additional delay periods 30 min following ketamine treatment compared to vehicle treated SD rats (Figure 5B). A significant main effect of group (*p* < 0.001, two-way ANOVA, *F*_1, 78_ = 19.11,) and delay interval (*p* = 0.001, two-way ANOVA, *F*_5, 78_ = 5.807) was observed. There was no strain x delay interaction (two-way ANOVA, *F*_5, 78_ = 1.113). *Post-hoc* analysis (Fisher's LSD test) revealed impairment of performance at three delay intervals: 11–15 s (*p* < 0.05), 21–25 s (*p* < 0.01), and 26–30 s (*p* < 0.01). Notably, 40% of WKY rats did not complete the DNMP task 30 min post-administration of ketamine. When delay periods were grouped into short (1–15 s) and long (16–20 s) delays (Figure 5E) a significant main effect of group (two-way ANOVA, *F*_1, 26_ = 10.31, *p* < 0.01) and delay interval (two-way ANOVA, *F*_1, 26_ = 9.39, *p* < 0.01) was observed. There was no strain x delay interaction (two-way ANOVA, *F*_1, 26_ = 1.257). *Post-hoc* analysis (Fisher's LSD test) revealed that ketamine treated WKY rats showed an impairment in correct responses in the long delay periods (*p* < 0.01) in comparison to SD rats.

### A Working Memory Deficit Was No Longer Observed in WKY Rats 24 h Post-administration of Ketamine

There was no significant difference in DNMP performance in WKY rats 24 h following ketamine treatment compared to vehicle treated SD rats (Figure 5C). No significant main effect of group (two-way ANOVA, *F*_1, 102_ = 0.7205) was observed. A significant effect of delay interval (*p* < 0.001, two-way ANOVA, *F*_5, 102_ = 6.289) was observed. There was no strain x delay interaction (two-way ANOVA, *F*_5, 102_ = 0.4388). All rats completed the task 24 h post-administration of ketamine. When delay periods were grouped into short (1–15 s) and long (16–20 s) delays (Figure 5F) a significant main effect of delay interval (two-way ANOVA, *F*_1, 26_ = 9.39, *p* < 0.01) was observed. No significant effect of group (two-way ANOVA, *F*_1, 34_ = 0.3475) was observed. There was no strain x delay interaction (two-way ANOVA, *F*_1, 34_ = 0.0845). *Post-hoc* analysis (Fisher's LSD test) revealed no significant difference in correct responses in WKY rats 24 h after ketamine treatment in comparison to SD rats.

## Discussion

The present study confirmed the depressive-like phenotype of WKY rats in several behavioral paradigms, including those related to the MDD symptoms of anhedonia and cognitive impairment. After establishing a ketamine dosing regimen that produced a clinically relevant plasma exposure profile, we showed that the rapid and sustained antidepressant-like effects of ketamine were accompanied by transient behavioral deficits. We also observed a biphasic alteration in performance in the DNMP working memory assay. Specifically, we showed: (i) anxiety-like behavior, anhedonia, depressive-like behavior and working memory impairment in WKY rats compared to SD rats; (ii) ketamine administered at 5 mg/kg, s.c. produced a plasma concentration similar to that observed in human plasma after administration of an efficacious dose in depressed patients (33); (iii) ketamine induced an antidepressant-like effect in the FST 30 min and 24 h after administration; (iv) 30 min after administration ketamine reduced LMA, USVs and induced a working memory deficit; (v) 24 h after administration the effects on LMA and USVs were no longer observed and ketamine improved working memory performance.

Anxiety-like behavior of WKY rats in the open field test has been reported previously (34). WKY rats show a generalized reduction in locomotor activity which may confound the interpretation of their behavior in the open field test, however, anxiety vulnerability has been confirmed in startle response compared to SD rats, representing a healthy control strain (35). This study also confirmed depressive-like behavior in the FST in WKY rats compared to SD rats (16). WKY rats were previously reported to show anhedonia-like behaviors (36) and we report, that WKY rats exhibited anhedonia in the FUST as indicated by an absence of “positive” 50 kHz USVs emitted during female urine exposure, compared to a significant increase in such calls emitted by SD rats under the same conditions. No difference between WKY and SD rats was observed with regard to non-evoked USVs emitted in the home cage, indicating no overall strain difference in USVs. Moreover, these data highlight the importance of coupling the FUST to USV recording when exploring hedonic behavior as previously suggested (22), since SD rats showed equal time sniffing female and male urine, yet only emitted 50 kHz vocalizations during female urine exposure. For the first time, we report that WKY rats exhibited deficits in the reward based DNMP task, an operant conditioning-based working memory assay assessing higher complexity cognitive functions compared to spatial memory tasks where WKY rats do not show deficits (37). Working memory deficits have been reported in MDD patients in cognitive tasks comparable to the DNMP task (38), thus validating WKY rats for studying cognitive abnormalities of translational value. WKY rats have also been shown to exhibit other translational depressive-like behaviors, including social avoidance (34) and altered sleep patterns (39).

WKY rats are proposed as a model of antidepressant resistance as SSRIs do not reverse depressive-like behavior in this model (16, 19). Here, we show that single administration of low doses of ketamine (1, 3, and 5 mg/kg, s.c.) reduced immobility in the FST 30 min post-administration, demonstrating ketamine's rapid antidepressant-like efficacy. Similar effects of low dose ketamine administered by the intraperitoneal route have been previously reported in male and female SD rats (40), and female WKY rats (21). The 1, 3, and 5 mg/kg doses of ketamine used in this study produced varying plasma ketamine concentrations 36 min post-administration, with 5 mg/kg producing a plasma exposure of 225 ng/ml. In a second pharmacokinetic experiment, the 5 mg/kg dose produced a plasma maximum concentration (C_max_) of 353 ng/ml at 30 min and a brain C_max_ of 1,620 ng/g at 15 min. Minimal levels were detected by 360 min post-administration. The plasma concentration achieved here with 5 mg/kg ketamine shows similarities with the clinically effective dose in MDD patients that produced a plasma C_max_ of 204 ± 102 ng/ml (33). This differs from that produced by the 10 mg/kg dose and intraperitoneal route of administration inducing antidepressant-like effects in mice, where an earlier plasma t_max_ of 10 min and higher C_max_ of ~600 ng/ml was reported (41). An earlier plasma t_max_ was also reported in rats when ketamine was administered by the intraperitoneal route (42). Taken together, this suggests 5 mg/kg s.c. ketamine administration in WKY rats is a more suitable method for producing clinically relevant plasma exposure. A differing concentration profile with respect to free brain ketamine was also evident between the WKY rat model in this study and mouse models employed by Zanos et al., where an ~35% lower C_max_ of 1,200 ng/ml was observed at 15 min post-administration (41). Thus, the 5 mg/kg, s.c. dosing regimen in WKY rats represents an optimal method to produce a clinically relevant plasma racemic ketamine concentration. However, further investigation is required to assess the suitability of the regimen for modeling clinically relevant plasma enantiomer and metabolite profiles (43), that could be significant in the pharmacology of ketamine and its antidepressant efficacy (44).

The 5 mg/kg, s.c. dose of ketamine was used for subsequent behavioral analysis in the present study and, in addition to its rapid antidepressant-like effects in the FST 30 min post-administration, a sustained effect at 24 h was also observed. The 24 h time point may be the optimal time for assessing the antidepressant-like effects of NMDA receptor antagonists in preclinical models. MK-801, a drug of this class that does not show clinical efficacy, is efficacious acutely (1 h) in preclinical models whereas the clinically efficacious ketamine showed sustained effects at 24 h in animal models (41). The acute effects of ketamine in the FST at 30 min reported in the present study were accompanied by a significant decrease in locomotion and this was not observed at 24 h. Transient increases in motor behavior have been well-documented in rodent models following acute ketamine treatment, with dose-dependent impairments in motor coordination identified (41, 45). The temporal hypolocomotion reported here in rats was in contrast to hyperlocomotion observed in mice, albeit induced at higher 10–30 mg/kg doses (45, 46). However, this finding may show similarities to the acute sedative effects reported clinically with both ketamine and esketamine (7, 47). In addition, we observed an almost complete inhibition of vocalizations at the 50 kHz frequency 30 min post-ketamine administration, and similar to the effects on locomotion, no difference was seen between the vehicle and ketamine treated groups by 24 h. A reduction in 50 kHz vocalizations has been reported in SD rats 30 min following treatment with 20 mg/kg but not lower doses of ketamine (48), suggesting a possible heightened sensitivity of WKY rats to ketamine. Although, the intraperitoneal route of administration used by Popik et al. may be significant considering the altered pharmacokinetic profile vs. the subcutaneous route used in the current study. Vocalizations at 50 kHz in rats are linked to positive affect (23), social behaviors (49) and communication (24). The acute inhibition of 50 kHz USVs reported here may model aspects of transient dissociation routinely reported in the clinic following ketamine treatment using the Clinician-Administered Dissociative States Scale (50), of which communication and social behaviors feature (51). Reduced social interaction following ketamine treatment has been previously reported in mice (45) and rats (48). Furthermore, reduced 50 kHz USVs in rats represent a method for predicting the aversive effects of drugs (52).

Biphasic effects of ketamine on working memory performance were also observed in this study. Vehicle treated WKY rats showed a deficit in the DNMP task in comparison to SD rats and this deficit is accentuated in WKY rats 30 min after ketamine administration. Specifically, the deficit was more pronounced at the longer delay intervals representing 16–30 s, the period of the task that is primarily hippocampal-dependent (53). Acute cognitive deficits induced by ketamine in mouse passive avoidance and rat novel object recognition tests have been shown previously (48). Such acute deficits may model aspects of the transient dissociation consistently reported in clinical studies of TRD and MDD patients (47) and show similarities to the acute impairment in cognitive function reported 40 min but not 2 h after esketamine administration in healthy participants (54). We report that ketamine has an opposing effect on working memory at 24 h post-administration where performance in WKY was undistinguishable from SD rats. This pro-cognitive effect 24 h following treatment has not been observed in other preclinical memory assays, including the novel object recognition task (45), suggesting that this may be specific to working memory. Significantly, working memory deficits are one of the most commonly reported cognitive symptoms in MDD patients and often persist following treatment and during remission (55). The majority of antidepressant drugs currently on the market are not approved for treating the cognitive symptoms of MDD, with the exception of vortioxetine that has demonstrated cognitive enhancing effects (56). Our data in WKY rats suggest ketamine as another potential treatment warranting investigation, and this is in line with clinical evidence of potential long-term pro-cognitive effects of ketamine in TRD patients (57).

This finding may also be of significance to assessing antidepressant efficacy in rodents. It has been suggested that in preclinical models only sustained effects on behavior when ketamine is eliminated from the system are related to its antidepressant efficacy as acute effects may be related to off target side effects (41). In line with this, in addition to a sustained reduction in depressive-like behavior in the FST 24 h post-administration of ketamine, we report an acute effect in the FST at 30 min when side effects such as reduced LMA and USVs were observed. By contrast, working memory in the DNMP task was further impaired compared to SD rats acutely at 30 min, while at 24 h a deficit in WKY rats compared to SD rats was no longer observed, thus clearly differentiating between acute and sustained effects of ketamine. Furthermore, immobility in the FST has been used for decades as a measurement of both depressive-like behavior and the efficacy of antidepressant drugs in rodents (17, 58) and is routinely used to study ketamine (8). Despite its utility as a behavioral pharmacodynamic assay, the validity and translational capacity of the FST with respect to modeling MDD is often questioned (59, 60). The assessment of behavioral endophenotypes directly related to MDD symptoms, such as working memory deficits in the DNMP task reported here, are proposed as important methods for future antidepressant drug discovery (61). Taken together, this suggests that the WKY rat model of MDD in combination with the DNMP assay may represent an optimal method for evaluating novel antidepressant drugs with a mechanism of action similar to ketamine.

## Conclusion

This study confirmed the depressive-like behavioral phenotype of WKY rats. Subcutaneous administration of 5 mg/kg ketamine in WKY rats represents an optimal method for modeling plasma drug exposure produced with the clinically effective dose in MDD patients. We show that transient side effects similar to those observed in the clinic accompany the acute (30 min) but not the sustained (24 h) reversal of depressive-like behavior in the FST following ketamine treatment. The acute impairment in working memory in the DNMP task induced by ketamine is followed by an enhancement at 24 h and may represent a novel and translational assay for exploring antidepressant efficacy.

## Data Availability Statement

The raw data supporting the conclusions of this article will be made available by the authors, without undue reservation.

## Ethics Statement

The animal study was reviewed and approved by Trinity College Dublin, Animal Research Ethics Committee.

## Author Contributions

JP, CM, and MB conceived, designed the experiments, analyzed, and interpreted the data. JP, CM, FD-D, and JR performed the experiments. JP and CM wrote the manuscript. ES and NU provided critical revision. All authors contributed to the article and approved the submitted version.

## Conflict of Interest

CM, FD-D, NU, ES, and JP are employees of Transpharmation Limited. JR and MB are former employees of Transpharmation Limited. The authors declare that this study received funding from Transpharmation Limited and H. Lundbeck A/S. The study design, analysis and interpretation of data, writing of the manuscript and the decision to submit for publication represent the views of the authors and not necessarily that of the funding sources.
